# Genomic analysis reveals selection in Chinese native black pig

**DOI:** 10.1038/srep36354

**Published:** 2016-11-03

**Authors:** Yuhua Fu, Cencen Li, Qianzi Tang, Shilin Tian, Long Jin, Jianhai Chen, Mingzhou Li, Changchun Li

**Affiliations:** 1 Key Lab of Agriculture Animal Genetics, Breeding, and Reproduction of Ministry of Education, College of Animal Science and Technology, Huazhong Agricultural University, Wuhan, 430070, PR China; 2 Institute of Animal Genetics and Breeding, College of Animal Science and Technology, Sichuan Agricultural University, Chengdu, 611130, PR China

## Abstract

Identification of genomic signatures that help reveal mechanisms underlying desirable traits in domesticated pigs is of significant biological, agricultural and medical importance. To identify the genomic footprints left by selection during domestication of the Enshi black pig, a typical native and meat-lard breed in China, we generated about 72-fold coverage of the pig genome using pools of genomic DNA representing three different populations of Enshi black pigs from three different locations. Combining this data with the available whole genomes of 13 Chinese wild boars, we identified 417 protein-coding genes embedded in the selected regions of Enshi black pigs. These genes are mainly involved in developmental and metabolic processes, response to stimulus, and other biological processes. Signatures of selection were detected in genes involved in body size and immunity (*RPS10* and *VASN*), lipid metabolism (*GSK3*), male fertility (*INSL6*) and developmental processes (*TBX19*). These findings provide a window into the potential genetic mechanism underlying development of desirable phenotypes in Enshi black pigs during domestication and subsequent artificial selection. Thus, our results illustrate how domestication has shaped patterns of genetic variation in Enshi black pigs and provide valuable genetic resources that enable effective use of pigs in agricultural production.

From early domestication to modern breeding practices, artificial selection for agriculturally important traits has shaped the genomes of domestic pigs. Guided by the reference genome of the domestic Duroc pig, considerable attempts have been made to unveil the genetic bases of phenotypes by using whole-genome wide SNP chip and resequencing approaches^1^,^2^,^3^,^4^. Wilkinson *et al*. found genomic regions associated with coat colour and ear morphology using Porcine60 K SNP chip^4^. Li *et al*. revealed strong signatures of selection in Berkshire affecting disease resistance, pork yield, fertility, tameness and body length^1^. Rubin *et al*. determined three selected genes that associate with body length in European commercial breeds^2^. Wang *et al*. revealed evidence of artificial selection of coat colour and reproductive traits in Chinese Tongchen pigs^3^.

Enshi black pigs, which comprise a typical native black breed in China, are best known for their fat storage ability and cold-wet tolerance. They are mainly raised in mountainous regions with an average altitude of 800 m above sea level in southwest China^5^ (Enshi Tujia minority and Miao minority autonomous prefecture) (Fig. 1). Enshi black pigs have undergone fewer systematic genetic improvement programs compared to other breeds since the 17^th^ century and are characterized by their average-sized head, concave and wrinkled face, well-developed limbs, concave back, tilted haunch and big belly^6^. Although they have adapted to the harsh local environment, Enshi black pigs are markedly different from wild boars, especially in terms of fat storage ability. Historically, Enshi black pigs have been intensively used in dry-cured ham production, leading to preferential selection based on their meat and fat quality traits, such as lean percentage, fat content, and eating quality^5^,^6^ (juiciness, flavor, tenderness, pink hue and heavy marbling) (Fig. 2).

To identify signatures of selection resulting from domestication, we performed whole-genome pooled resequencing of three representative populations of Enshi black pigs (~202.21 Gb in total; ~24.07× coverage per population). Together with 96 publicly available genomes of pig breeds found in Asia (13 Chinese wild boars, 10 Korean wild boars and 73 Chinese domestic pigs) and using a total of ~4.00 trillion bases of resequencing data^7^,^8^,^9^,^10^,^11^ (Supplementary Table S1), we conducted a comprehensive analysis of genetic diversity and sought to identify genomic regions under selection in the Enshi black pig.

## Results and Discussion

### Sequencing, SNP calling and annotation

Pooled paired-end sequencing of the three populations of Enshi black pigs generated 70.64, 67.86 and 63.71 Gb data for the genomes of the Lvcongpo (LCP), Yetinglu (YTL) and Zhongbaozhen (ZBZ) populations, respectively. To accurately detect genomic footprints left by selection, we downloaded the publicly available genome data of 13 Chinese wild boars (Supplementary Table S1). To reduce possible bias resulting from differences in sequencing coverage between the Enahi black pigs (~24.07× coverage per population) and the Chinese wild boars (~13.19× coverage per individual) and to accurately detect genomic footprints left by selection, we randomly selected ~4.80 Gb of high-quality sequencing data from each individual of 13 Chinese wild boars^7^,^9^,^10^, which simulated pooled sequencing of a Chinese wild boar population, yielding a total of 61.82 Gb of sequencing data.

A total of 248.91 Gb high-quality sequencing data from three Enshi black pig populations and a Chinese wild boar population were aligned against the reference pig genome (Sscrofa10.2) using BWA (v0.7.8)^12^. For each population, ~94.29% of the high-quality reads were mapped to the reference pig genome, of which ~81.82% were uniquely mapped, with comparable genome coverage (~13.32× to 15.70×) between the three populations of Enshi black pig and one population of Chinese wild boar (Supplementary Table S2).

We identified ~14.09 M SNPs in four populations that were concurrently cataloged by two currently dominant algorithms (i.e. SAMtools^13^ and GATK^14^), which accounted for 88.12% and 86.76% of SNPs that were identified by SAMtools and GATK, respectively, and were used for subsequent analyses (Supplementary Figure S1, Supplementary Table S3). In total, 0.28–0.30 M (2.01–2.06%) SNPs were considered novel based on their absence in the pig dbSNP (Build 143) database (Supplementary Table S3).

Compared with the 60.92% SNPs (~10.70 M of 17.57 M) that were shared between the Enshi black pigs and Chinese wild boar, more than three-quarter of SNPs (~12.06 M of 15.98 M, or 75.56%) were shared among three Enshi black pig populations (Supplementary Figure S2), which indicated substantial genomic similarity between Enshi black pigs from the three major distributed locations (Fig. 3). Furthermore, the neighbor-joining tree of the four populations revealed two clusters (three Enshi black pig populations and a Chinese wild boar population) (Supplementary Figure S3a). This finding is consistent with the low diversity among the three Enshi black pig populations, as revealed by the phylogenetic analysis of complete mitochondrial DNA sequences of Eurasian pigs (Supplementary Figure S4).

We identified 113,864 non-redundant coding SNPs from four populations, of which 91,353, 91,403, 91,639 and 79,155 are from the LCP, YTL, ZBZ and wild boar populations, respectively (Supplementary Figure S5). We detected 76,168 shared coding SNPs in three Enshi black pig populations, and 25,816 nonsynonymous nucleotide substitutions (25,595 missense, 183 stop gain and 38 stop loss) in 8,301 genes (Table 1). The top 1,053 genes containing the highest number (*n* ≥ 7) of nonsynonymous SNPs were mainly over-represented in the highly variable sensory perception categories (Supplementary Table S4), which reflects the strong reliance of pigs on their sense of smell while scavenging for food and other odor-driven behavior.

### Selective sweep analysis

We analyzed the pooled sequences for selective sweeps in Enshi black pigs by searching for genomic regions with excess homozygosity and/or increased genetic distance to Chinese wild boar (*F*_ST_). We identified 1,051–1,078 regions (195.15 M to 198.16 M) in the Enshi black pig genome with extremely low levels of heterozygosity (*Z*(*H*_P_) < −2) and 1,043–1,067 regions (208.94 M to 210.96 M) with strongly elevated *F*_ST_ values (*Z*(*F*_ST_) > 2). In total, 463–487 unique candidate domestication regions (CDRs) (from 81.77 M to 87.52 M) containing 858–1,046 genes were identified in three populations by focusing our analysis of putatively selected regions on those that fall at least two standard deviations away from the mean (*Z*(*H*_P_) < −2 and *Z*(*F*_ST_) > 2, Supplementary Figure S6–8). The large overlap between the *H*_P_ and *F*_*ST*_ regions indicated that the two statistical methods detected the same events. SNPs from these CDRs formed two distinct clusters (i.e. three Enshi black pig populations and a Chinese wild boar population) (Supplementary Figure S3b).

We detected 417 candidate selected genes (CSGs) in 185 CDRs (30.91 M, Supplementary Data S1), which were shared among the three populations. Most of the selected genes presented a lower degree of haplotype sharing between the Enshi black pigs and the Chinese wild boar breeds, and highly similarity of haplotypes among three Enshi black pig populations (Fig. 4). These CSGs were mainly overrepresented in developmental processes (hemopoiesis, *P* = 1.39 × 10^−11^; cell differentiation, *P* = 2.27 × 10^−9^; system development, *P* = 3.36 × 10^−2^), metabolic processes (regulation of phosphate metabolic process, *P* = 7.68 × 10^−10^; protein phosphorylation, *P* = 3.47 × 10^−7^; biosynthetic process, *P* = 4.04 × 10^−4^; phosphate-containing compound metabolic process, *P* = 1.44 × 10^−4^; and cellular protein modification, *P* = 8.34 × 10^−3^), and response to stimulus (natural killer cell activation, *P* = 4.76 × 10^−11^; response to stress, *P* = 1.63 × 10^−4^) (Table 2). These rapidly evolved genes in Enshi black pigs may be responsible for the dramatic phenotypic changes that are of economic value, such as growth rate, fat storage ability and disease resistance. Six CSGs were found to be related to lipid transport and metabolism (*CYP2F1*, *PISD*, *TKTL2*, *SEC14L5*, *PRSS33*, and *ACSF2*), which may have resulted from selection driven by a demand for energy-rich food during the development of fatty Enshi black pigs in China. Consistent with previous reports^1^,^15^ on domesticated pigs, the rapid evolution of 29 genes involved in immune-related processes (Table 2) may have contributed to the human-desirable selection of disease resistance. In addition, a concise FI network was constructed based on the 417 CSGs, with *UBC*, *JAK3*, *RAC1*, *RELA* and *MAPK1* as the central node genes (Supplementary Figure S9). The central node genes were those involved primarily in cellular and immune-related processes.

### Domestication genes

To identify the key genes that play an important role in shaping the domestication of Enshi black pigs, we performed statistical analysis (Student’s *t* test) using the identity score (IS) of the two conditions (IS between the Enshi black pigs and the Chinese wild boar breeds or IS among three Enshi black pig populations). We then selected top 10 genes (*ITPR3*, *RPS10*, *ERF*, *INSL6*, *ENSSSCG00000014230*, *TBX19*, *SFT2D2*, *VASN*, *GSK3A*, and *ORMDL1*) with the lowest *P* value for further study.

To analyze the signs of selection in detail, we detected SNPs from the regions spanning these genes with highly significant effects (SNPs located in untranslated regions (UTRs), exon, and downstream/upstream of the gene). We found 34 SNPs with significantly different mutation frequency between Enshi black pigs and Chinese wild boars (Fig. 5) in *RPS10*, *GSK3A*, *INSL6*, *TBX19*, and *VASN*. These SNPs may affect protein coding, gene splicing, transcription factor binding, and regulation of gene expression directly or indirectly. Furthermore, we genotyped the 34 SNPs in 15 pig breeds that represent a wide range of Chinese domestic pig populations (73 individuals), 13 Chinese wild boars, and 10 Korean wild boars^11^ (Fig. 6). The results demonstrated strong signatures of selection at these loci across Chinese domestic pigs that are used for pork production (i.e. muscle growth and adipose deposition). By combining the mutation frequency and genotyping results, we found that five genes were extremely different between the domestic pig breeds and Chinese wild boars. These genes may be responsible for the marked phenotypic changes produced by domestication of Enshi black pigs.

Among these five genes, two genes may be associated with body size and immunity. The first gene is ribosomal protein S10 (*RPS10*), which harbors three mutations in the 3′ UTR, and has also been reported to be commonly mutated in Diamond-Blackfan Anemia^16^,^17^. This gene provides instructions for producing ~80 different ribosomal proteins, which are vital components of cellular structures called ribosomes^18^. *RPS10* may be associated with body height, body length, and longissimus muscle weight in pigs^19^,^20^. Interestingly, a previous genome-wide association study (GWAS) also showed that *RPS10* may be associated with limb bone length (which is associated with body height and body length)^21^. Our study confirmed this conclusion. The second gene is Vasorin (*VASN*), which has three nonsynonymous mutations. This gene may be involved in modulating arterial response to injury by inhibiting the TGF-*β* signaling pathway^22^,^23^,^24^. *VASN* is highly expressed in vascular smooth muscle cells (hence the name) and the developing skeletal system^25^. This expression pattern indicates that *VASN* may indirectly influence the body size of pigs during embryonic development.

As a typical meat-lard pig breed, Enshi pigs exhibit strong selection signals in the obesity-related gene glycogen synthase kinase 3 (*GSK3*). *GSK3* is a constitutively active, proline-directed serine/threonine kinase that participates in a number of physiological processes that range from glycogen metabolism to gene transcription^26^. The gene exists in two isoforms, *GSK3A* and *GSK3B*^27^. *GSK3A* knockout experiment in mice displayed improved glucose tolerance in response to glucose load and elevated hepatic glycogen storage and insulin sensitivity^28^, which may result in obesity^29^,^30^. Association analysis revealed that the *GSK3A Hin*1I and *Msp*I polymorphisms were significantly associated with loin muscle area, average back fat thickness, thorax-waist fat thickness, and buttock fat thickness^31^. In addition, the gene could influence the muscle-to-meat process via glycolysis, reduced pH, and pale color^32^,^33^. The selection of the *GSK3* gene may contribute to enhanced fat storage ability and relatively high intramuscular adipose content.

The remaining two genes were involved in developmental processes and male fertility. *TBX19* (known as *TPIT*) is a member of the T-box family of transcription factors, which are involved in the regulation of developmental processes^34^. Numerous reports demonstrated that mutation in this gene may isolate deficiency of pituitary POMC-derived ACTH^35^,^36^,^37^,^38^. ACTH deficiency is characterized by adrenal insufficiency symptoms, such as weight loss, lack of appetite, obesity, hypocortisolism, and low blood pressure^39^,^40^. Nonsynonymous mutations of this gene may exist in Tongcheng pigs^3^, and a strong selection signal was detected in all Chinese native pig in our analysis. These findings suggested that the selection of this gene exerted a comprehensive influence on all Chinese indigenous pigs. The final gene, insulin-like 6 (*INSL6*), is a member of the insulin superfamily, which comprises a group of structurally related proteins^41^. *INSL6* is predominantly expressed in the testis, and it is necessary for the progression of spermatogenesis. Deficiency in *INSL6* will cause varying levels of male infertility^42^,^43^,^44^,^45^,^46^.

These positively selected genes may serve as important genetic foundation of the evolutionary scenarios triggered by artificial selection for agricultural production of Enshi black pigs. Further studies are required to define the signaling cascades associated with these genes and their regulatory mechanisms in order to facilitate a better understanding of their roles in the formation of economically important native breeds.

## Conclusions

This study detected the genomic signatures that may have shaped the domestication of Enshi black pigs in China. The genes found to be positively selected in Enshi pigs are involved in crucial biological processes such as body size and immunity (*RPS10* and *VASN*), obesity (*GSK3*), male fertility (*INSL6*), and early development (*TBX19*). In addition, important mutations within these genes were also identified to enrich the pool of markers that can be used to further refine selection in these pigs in the future. Our research methods and findings should also be helpful in deciphering genomic footprints left by selection and domestication in other livestocks^47^,^48^,^49^.

## Materials and Methods

### Ethics statement

Animals care and all the experimentation in this study were carried out in accordance with the pre-approved guidelines from Regulation Proclamation No.5 of the Standing Committee of Hubei People’s Congress, and all experimental procedures and sample collection methods were approved by the Institutional Animal Care and Use Committee of Huazhong Agricultural University, Wuhan, China (permit HZAUSW2015-0003).

### Animals and tissue collection

**Figure 1:**
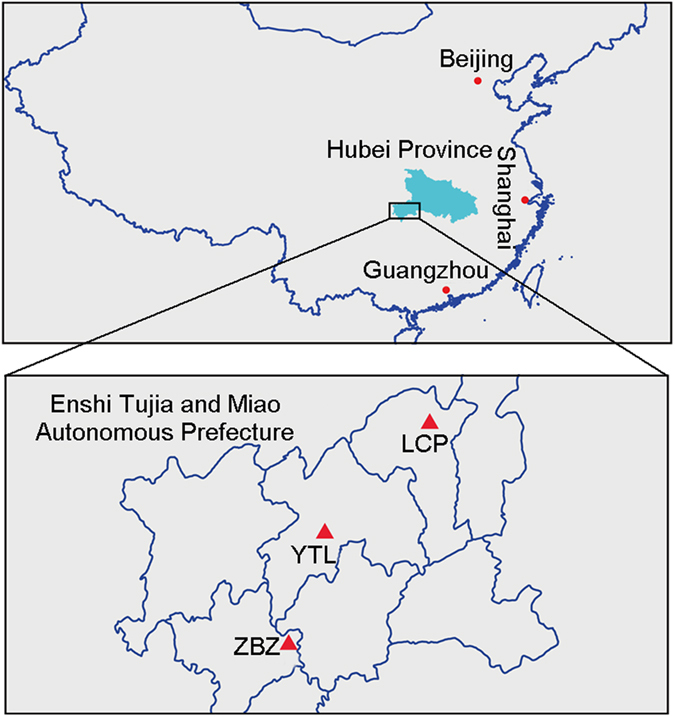
Geographical distribution of the Enshi black pigs used in our study. The map was created by using the “maptools” package (v0.839) of R software (v3.1.3, www.r-project.org).

**Figure 3:**
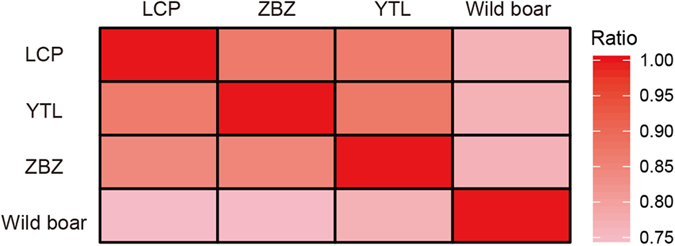
The ratio of shared SNPs between different populations.

**Figure 4:**
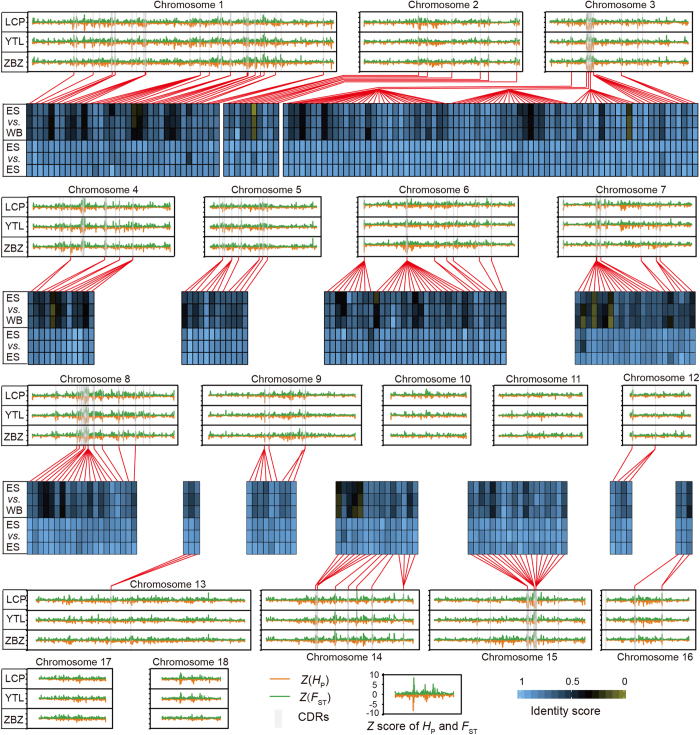
The candidate domestication regions and genes distribution along pig autosomes 1–18. The positive end of the *Z*(*F*_ST_) and the negative end of the *Z*(*H*_P_) distribution plotted for SNPs within each 50-kb window across the genome, the cut-off (|*Z*| > 2) used for extracting outliers, and the candidate domestication regions shared among three populations were marked with gray shadow. The degree of haplotype sharing for the candidate selected genes (CSGs) were achieved in pairwise comparisons. Boxes to the left indicate the comparison presented on that row (ES, Enshi black pig; WB, Chinese wild boar). Heatmap colors indicate identity scores (IS). LCP, Lvcongpo population; YTL, Yetinglu population; ZBZ, Zhongbaozhen population.

**Figure 5:**
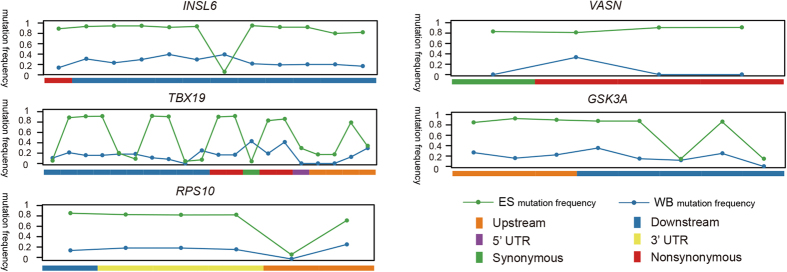
The difference of mutation frequency between Enshi black pig and Chinese wild boar. The mutation frequency in Enshi black pig is the average of three Enshi populations.

**Figure 6:**
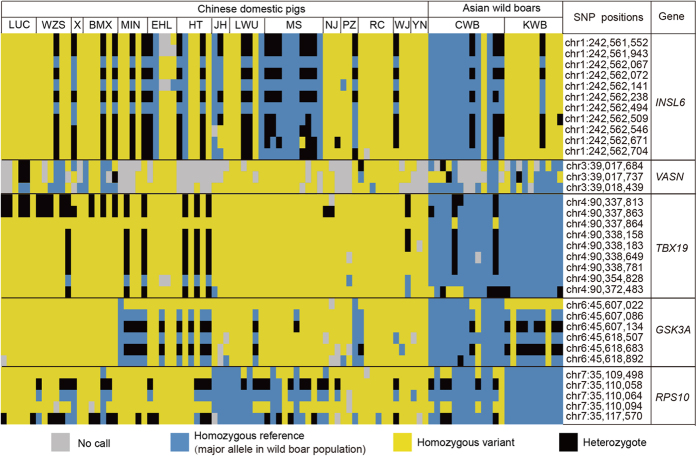
The genotyping results of Asian wild boars and a diverse panel of Chinese domestic pigs. Results from genotyping Asian wild boars and a diverse panel of Chinese domestic pigs for SNPs in the putative selective sweeps containing *INSL6*, *VASN*, *TBX19*, *GSK3A*, and *RPS10*. The plot is sorted by breeds but has not been clustered by individuals or by breeds. LUC, Luchuan; WZS, Wuzhishan; X, Xiang; BMX, Bamaxiang; MIN, Min; EHL, Erhualian; HT, Hetao; JH, Jinhua; LWU, Laiwu; MS, Meishan; NJ, Neijiang; PZ, Penzhou; RC, Rongchang; WJ, Wujin; YN, Yanan; CWB, Chinese wild boar; KWB, Korean wild boar.

Three representative populations of Enshi black pig were raised in counties of Enshi Tujia and Miao Autonomous Prefecture in Hubei Province, China (LCP, YTL, and ZBZ; Fig. 1). Blood samples were obtained from 75 female individuals (31 from the LCP, 32 from the YTL, and 12 from the ZBZ).

### Sequencing data

For each population, DNA samples from 12–32 individuals were pooled in equimolar quantities that were used to construct pair-end sequencing libraries with an insert size of 300 bp. The libraries were sequenced by Illumina Hiseq 4000 (Illumina, San Diego, CA, USA) with 150 bp paired-end reads according to the manufacturer’s instructions. We also downloaded 96 publicly available pig genomes in Asia (Supplementary Table S1), of which 13 Chinese wild boars were used to test for differentiation and possibly selection, and 10 Korean wild boars and 73 Chinese domestic pigs were used to investigate the patterns of selected loci.

To avoid reads with low-quality, we removed the following types of reads: (a) reads with ≥10% unidentified nucleotides (N); (b) reads with >10 nt aligned to the adapter, allowing ≤10% mismatches; and (c) reads with >50% bases having phred quality <5; and (d) putative PCR duplicates generated by PCR amplification in the library construction process. After the low-quality reads were excluded, the remaining high-quality reads were aligned against the Sscrofa10.2 reference sequence by using Burrows-Wheeler Aligner (BWA, v0.7.8), with the BWA command of “mem -t 4 -k 19 -M -w 200”. The uniquely aligned reads with ≤5 mismatches were used for SNP calling.

### SNP calling and variation annotation

To obtain highly confident SNPs, we employed both SAMtools (v0.1.19) and GATK tool (v3.3) variant calling pipelines to process each pool of samples respectively. In SAMtools, base calling was conducted by using the “mpileup” command and the “-q 1 -C 50 -S -D -m 2 -F 0.002” parameters of SAMtools. The “view” command of BCFtools was used to convert the BCF files to VCF files. The VCF files were then filtered by the “vcfutils.pl” script with the use of the “varFilter -Q 20 -d 4 -D 1000” option and vcftools using the “–thin 4” option, and high-quality SNPs (coverage depth ≥4 and ≤1000, RMS mapping quality >20, the distance between adjacent SNPs ≥5 bp) were retained for subsequent analysis. For GATK, the settings were used according to the GATK best practice online documentation. Results that were obtained by the two pipelines were compared using the BEDTools (v2.21.0) “intersectBed” module^50^, and only concordant variations were processed further.

To determine novel variants in our sequence data, we compared the identified SNPs with the dbSNP (Build 143) data using BEDTools and annotated the detected genetic variants using ANNOVAR^51^. Gene ontology analysis was performed by the web-based software PANTHER^52^. Functional interaction network of CSGs was performed by the Reactome FI Plugin in the Cytoscape software environment^53^.

### Selective-sweep analysis

For each population, we used allele counts at variable sites to identify signals of selection in 100 kb windows (with a step size of 50 kb) through two approaches: for each window, we calculated (1) the average pooled heterozygosity, *H*_P_ , and (2) the average fixation index, *F*_ST_, between Enshi black pig and Chinese wild boar. We calculated *F*_ST_ using PoPoolation2^54^ and *H*_P_ as follows:


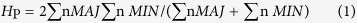


where nMAJ is the most frequently observed allele, and nMAJ is the least frequently observed allele.

Putatively selected regions in each group were located by extracting windows from the tails of the *Z*-transformed *H*_P_ and *F*_ST_ distributions by applying a threshold of two standard deviations (*P* ≤ 0.05, Z(*H*_P_) < −2, *Z*(*F*_ST_) > 2). We disregarded the selection signals on chromosome X because of the extremely low rate of recombination^55^ and ancient interspecies introgression^7^.

### Calculation of identity score (IS)

We calculated ISs to visualize haplotype sharing in pairwise comparisons at the selected genes. For each identified SNP, we determined the fraction of reads that corresponded to the reference genome allele, F, in each pig population. The IS values of individual SNPs were then calculated as IS = 1 − (|*F*_Population1_ − *F*_Population2_|) (2), with SNPs assessed only if a minimum of one read was obtained in each population. The IS value for a gene was the mean of all IS values observed in the gene for a specific comparison.

### Sequencing of the complete mitochondrial DNA (mtDNA) sequences

Ear tissue samples of five individuals were collected from the three counties (1 from LCP, 2 from YTL, and 2 from ZBZ; Supplementary Figure S4). DNA was isolated using a MicroElute Genomic DNA kit (OMEGA, USA). Nineteen pairs of primers (Supplementary Table S5) were used to amplify and sequence the complete mitochondrial DNA sequences. PCR reactions were performed using LA taq (TaKaRa, Dalian, China). PCR products were purified following agarose gel electrophoresis and then sequenced using the ABI 3730 DNA sequencer (Applied Biosystems, Foster City, CA, USA). Sequences of other representative domestic pigs and wild boars were downloaded from GenBank. The detailed information on the pig breeds are shown in Supplementary Table S6.

### Phylogenetic analysis

To explore the genetic relationship of the three populations of Enshi black pigs and other pig breeds, we used the five newly generated complete mtDNA sequences, together with 18 downloaded complete mitochondrial DNA sequences, to perform phylogenetic analysis; the neighbor-joining tree was constructed using MEGA (v5.0)^56^.

To explore the genetic relationship of the 4 pooled group, we also constructed another two phylogenetic trees by using SNPs in whole genome and SNPs in selected regions respectively. Homologous regions among different pig populations were identified and extracted by SNPhylo (v20160204)^57^. The corresponding SNPs in homologous regions were utilized to construct a neighbor-joining tree using MEGA.

## Additional Information

**Accession codes**: The genome resequencing reads have been deposited into the NCBI sequence read archive (SRA) under the accession SRP071318.

**How to cite this article**: Fu, Y. *et al*. Genomic analysis reveals selection in Chinese native black pig. *Sci. Rep*. **6**, 36354; doi: 10.1038/srep36354 (2016).

**Publisher’s note**: Springer Nature remains neutral with regard to jurisdictional claims in published maps and institutional affiliations.

## Supplementary Material

Supplementary Information

Supplementary Data S1

## Figures and Tables

**Figure 2:**
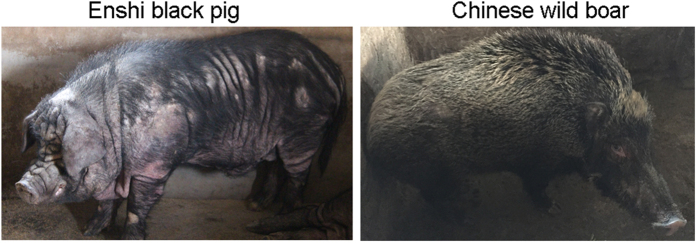
Image of Enshi black pig and Chinese wild boar.

**Table 1:** Summary and annotation of SNPs in Enshi black pigs and Chines wild boar.

Number of SNPs					
Category	LCP	YTL	ZBZ	ES	Wild boar
3’UTR	54,883	55,078	55,961	44,505	52,282
Intergenic	9,925,783	9,934,711	10,260,959	7,823,054	10,327,917
Splicing	402	421	430	351	392
Upstream	115,480	115,907	117,787	93,785	108,071
Exonic					
Stop gain	231	231	244	183	221
Nonsynonymous	30,849	30,819	31,005	25,595	26,568
Unknown	1,325	1,312	1,352	1,133	1,149
Synonymous	60,015	60,094	60,111	50,161	52,160
Stop loss	43	42	44	38	41
Downstream	113,563	113,845	116,124	90,862	110,907
5’UTR	11,869	12,041	11,920	10,034	8,700
Intronic	3,554,718	3,553,004	3,642,593	2,823,962	3,569,327

The package ANNOVAR was used to identify whether a SNP causes protein coding changes and the amino acids that are affected. LCP, Lvcongpo population; YTL, Yetinglu population; ZBZ, Zhongbaozhen population; Wild boar, Chinese wild boar population; ES, shared in three Enshi pig populations.

**Table 2:** Enriched gene ontology terms among CDR genes.

Term ID	Term description	Gene count	*P* value
GO-BP:0030101^‡^	Natural killer cell activation	14	4.76 × 10^−11^
GO-BP:0008283	Cell proliferation	14	8.05 × 10^−11^
GO-BP:0030097^†^	Hemopoiesis	15	1.39 × 10^−11^
GO-BP:0030154^†^	Cell differentiation	15	2.27 × 10^−09^
GO-BP:0019220*	Regulation of phosphate metabolic process	16	7.68 × 10^−10^
GO-BP:0006468*	Protein phosphorylation	23	3.47 × 10^−07^
GO-BP:0006950^‡^	Response to stress	20	1.63 × 10^−04^
GO-BP:0009058*	Biosynthetic process	21	4.04 × 10^−04^
GO-BP:0006796*	Phosphate-containing compound metabolic process	24	1.44 × 10^−04^
GO-BP:0006955	Immune response	15	7.26 × 10^−03^
GO-BP:0006464*	Cellular protein modification process	26	8.34 × 10^−03^
GO-BP:0048731^†^	System development	24	3.36 × 10^−02^

Enriched terms are symbol-coded to reflect relatedness in the ontology or functional proximity. *metabolic process; ^†^developmental process; ^‡^response to stimulus. For each term, gene count shows number of genes in CDRs.
