# Supplementary material for: Genomic analysis reveals selection in Chinese native black pig
Source: Sci Rep. 2016 Nov 3;6:36354. doi: 10.1038/srep36354 (PMC5093412; doi:10.1038/srep36354)
Supplement: Supplementary Information [file srep36354-s1.doc]

**Genomic analysis reveals selection in** **Chinese native black pig**

**Yuhua Fu^1,2^, Cencen Li^1^, Qianzi Tang^2^, Shilin Tian^2^, Long Jin^2^, Jianhai Chen^1^, Mingzhou Li^2*^, Changchun Li^1*^**

^1^ Key Lab of Agriculture Animal Genetics, Breeding, and Reproduction of Ministry of Education, College of Animal Science and Technology, Huazhong Agricultural University, Wuhan, 430070, PR China;

^2^ Institute of Animal Genetics and Breeding, College of Animal Science and Technology, Sichuan Agricultural University, Chengdu, 611130, PR China.

*Correspondence should be addressed to Changchun Li ([lichangchun@mail.hzau.edu.cn](mailto:lichangchun@mail.hzau.edu.cn)) or Mingzhou Li (mingzhou.li@163.com) .

**Supplementary files note:** supplementary Tables S1-S6 and supplementary Figures S1-S9.

The following supplementary material is available:


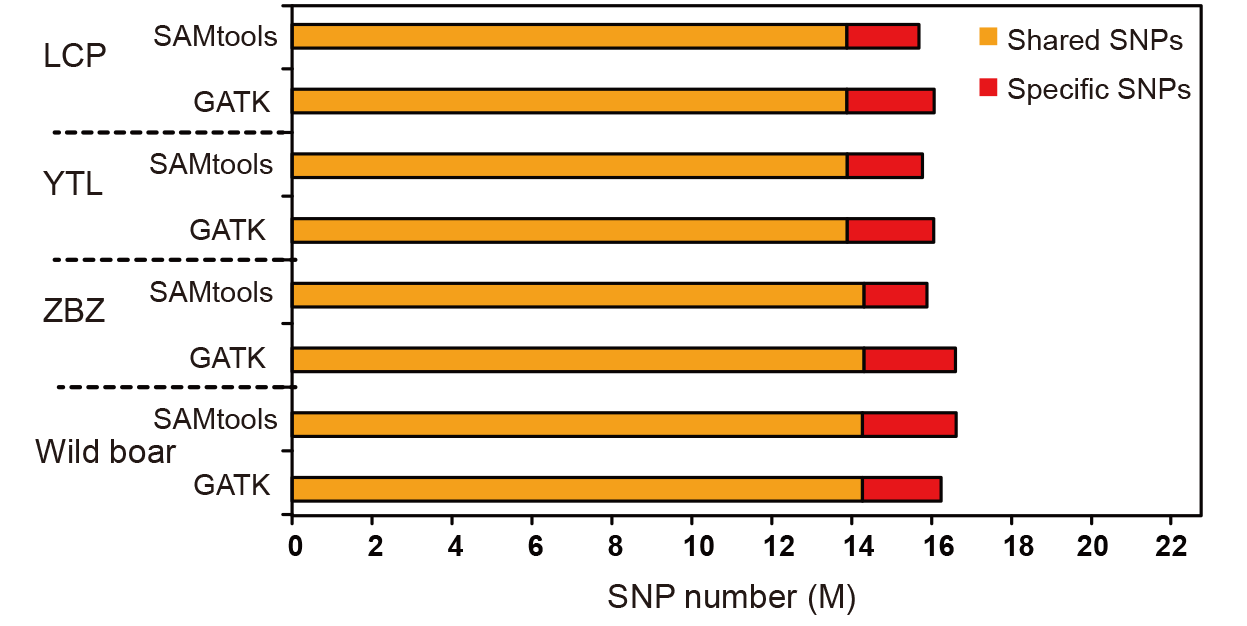


Supplementary Figure S1. Comparison of SNP calling between SAMtools and GATK tool. About 15.99 M and ~16.23 M SNPs for each population were detected using SAMtools and GATK, respectively. Of which ~14.09 M SNPs were concurrently identified by two algorithms, which account for 88.12% and 86.81% SNPs identified by SAMtools and GATK, respectively.


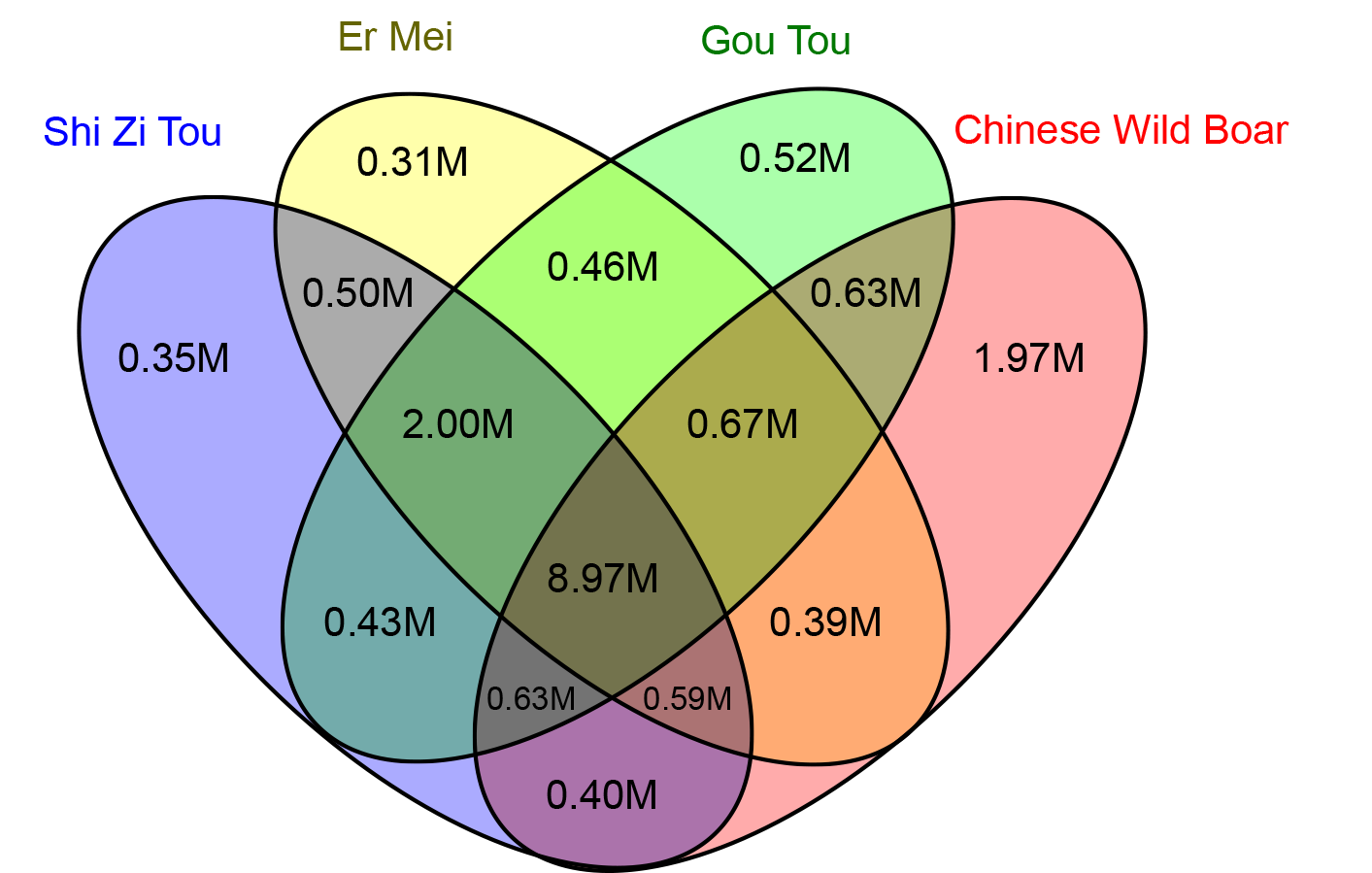


Supplementary Figure S2. Venn diagram of total SNPs in the Enshi black pigs.


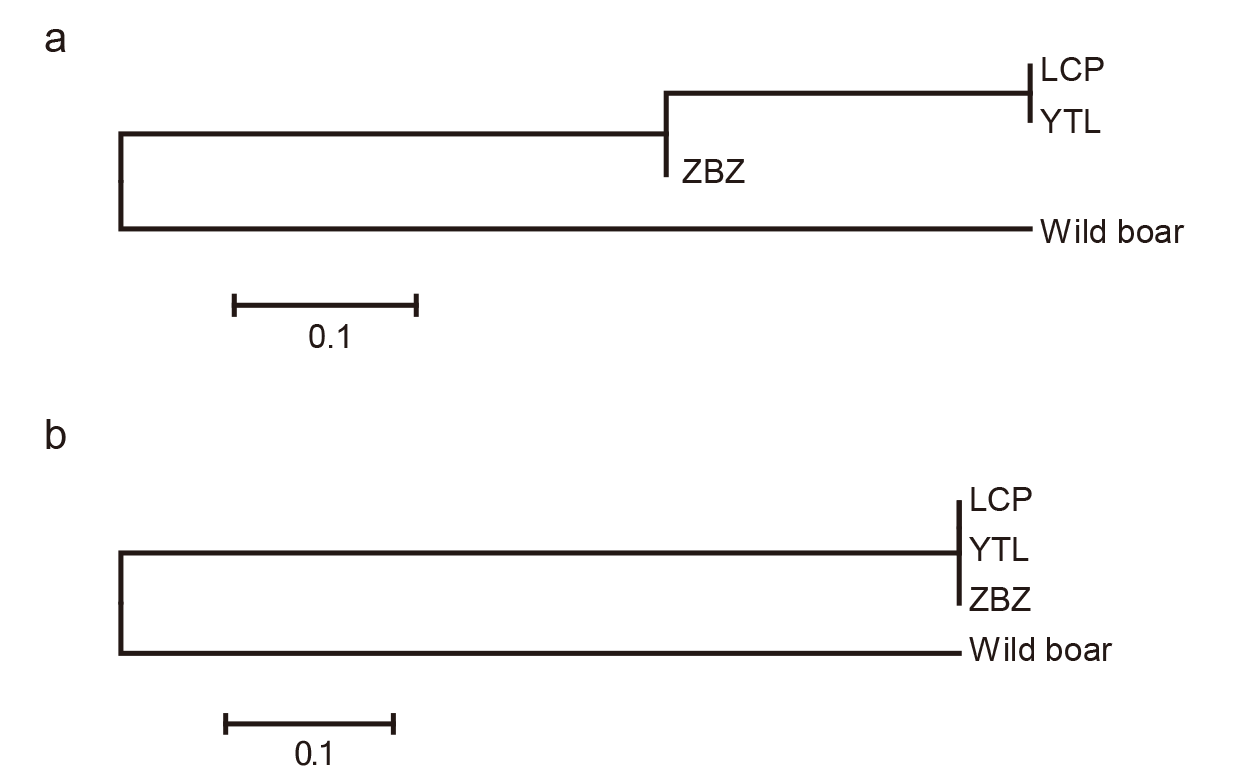


Supplementary Figure S3. Phylogenetic tree analysis of the four populations. (a) Phylogenetic tree analysis with whole genome SNPs; (b) Phylogenetic tree analysis with SNPs in CDRs. LCP, Lvcongpo population; YTL, Yetinglu population; ZBZ, Zhongbaozhen population; Wild boar, Chinese wild boar population;


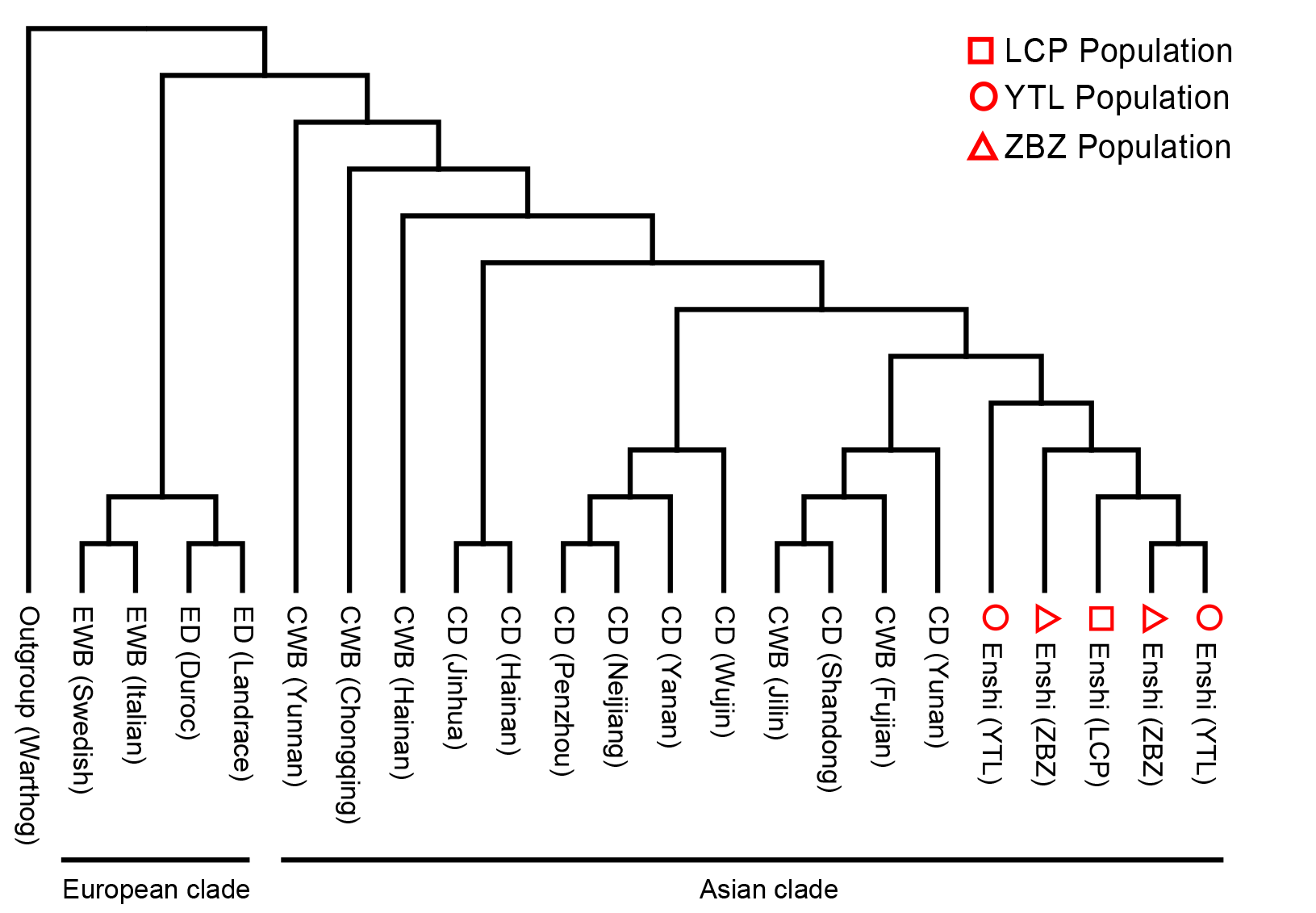


Supplementary Figure S4. The phylogenetic trees of 23 pigs. To explore the genetic relationship of the three populations of Enshi black pigs and other pig breeds, the determined 5 complete mtDNA sequences, together with 18 downloaded complete mtDNA sequences were used to perform phylogenetic analysis.


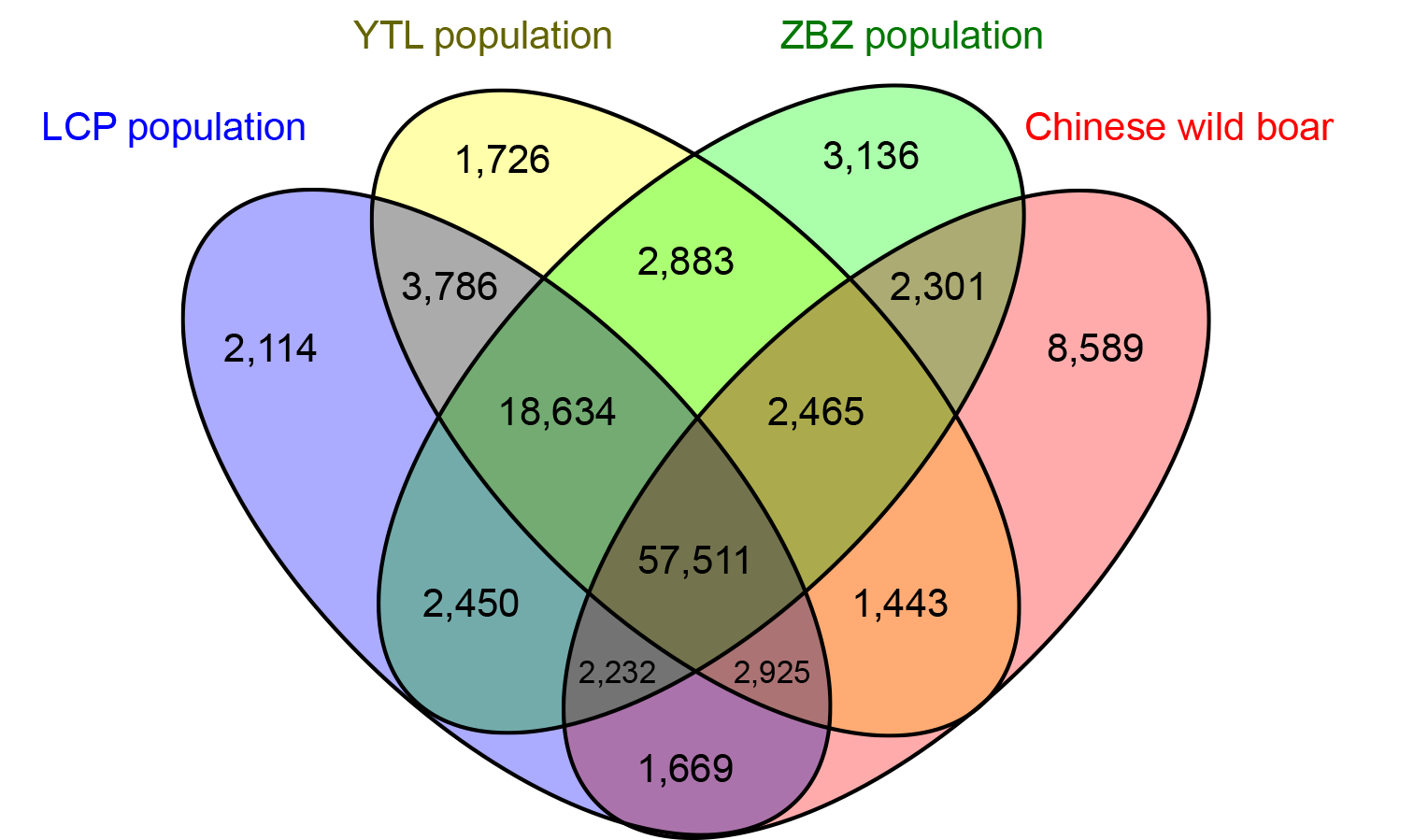


Supplementary Figure S5. Venn diagram of exonic SNPs in the sequenced animals.


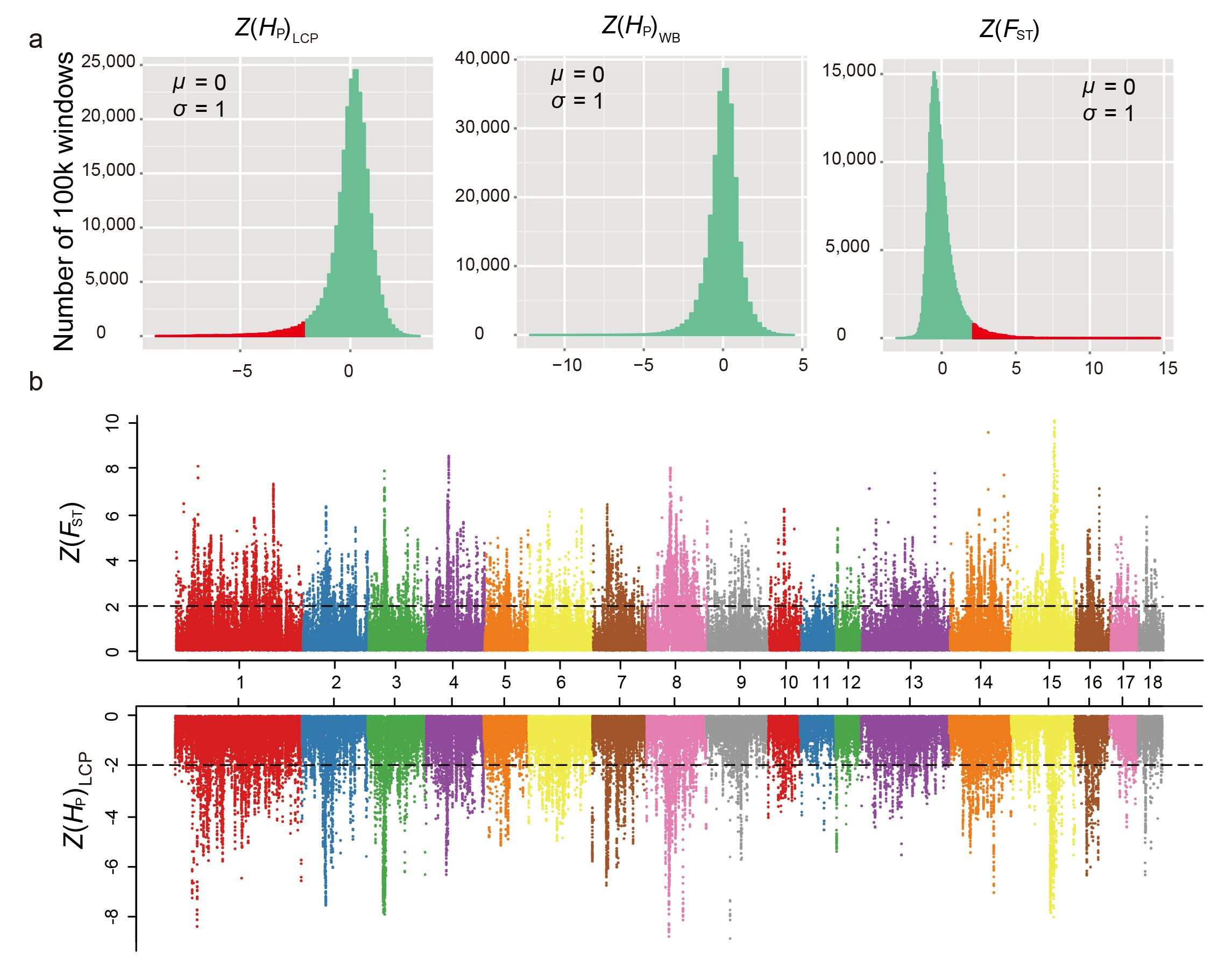


Supplementary Figure S6. Genome-wide selection analysis of Enshi black pigs in LCP population. a, Distribution of Z-transformed average pooled heterozygosity in LCP population (*Z*(*H*_P_) _LCP_), as well as average fixation index (*Z*(*F*_ST_)), for autosomal 100 kb windows (*σ*, standard deviation; *μ*, average). b, The positive end of the *Z*(*F*_ST_) and the negative end of the *Z*(*H*_P_) distribution plotted along pig autosomes 1-18. A dashed horizontal line indicates the cut-off (|*Z*| > 2) used for extracting outliers.


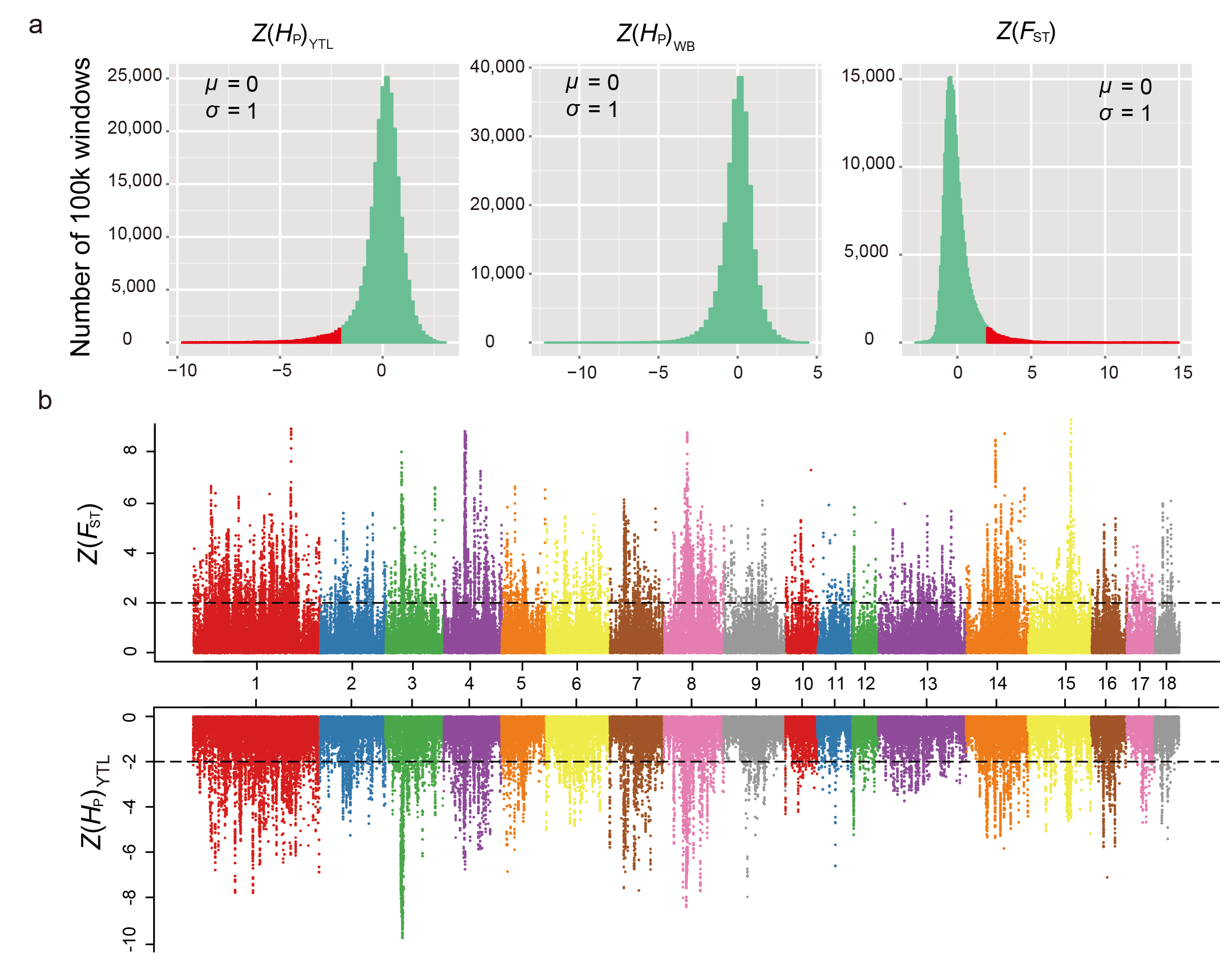
Supplementary Figure S7. Genome-wide selection analysis of Enshi black pigs in YTL population. a, Distribution of Z-transformed average pooled heterozygosity in YTL population (*Z*(*H*_P_)_YTL_) and wild boar (*Z*(*H*_P_)_WB_) respectively, as well as average fixation index (*Z*(*F*_ST_)), for autosomal 100 kb windows. b, The positive end of the *Z*(*F*_ST_) and the negative end of the *Z*(*H*_P_) distribution plotted along pig autosomes 1-18. A dashed horizontal line indicates the cut-off (|*Z*| > 2) used for extracting outliers.


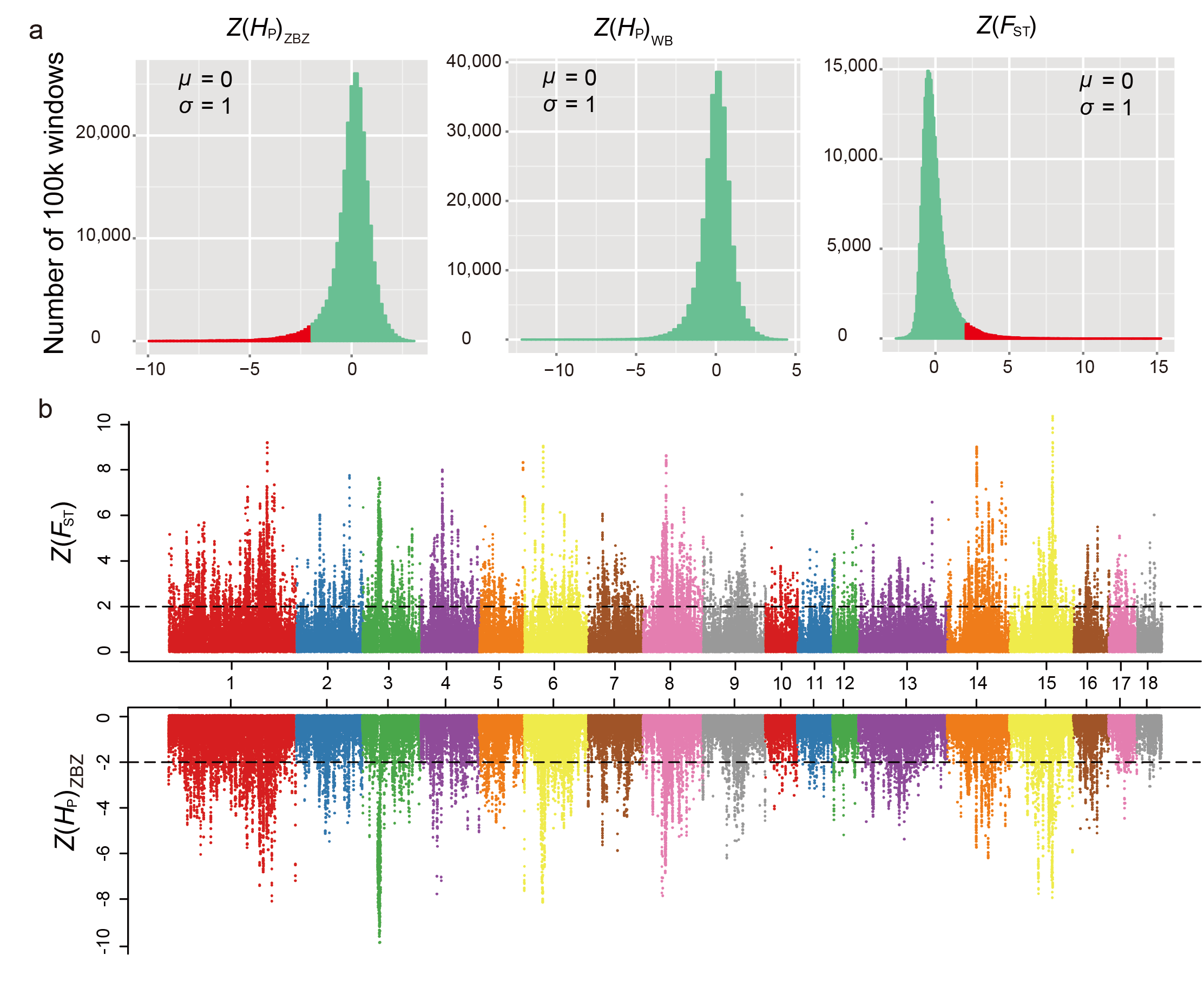


Supplementary Figure S8. Genome-wide selection analysis of Enshi black pigs in ZBZ population. a, Distribution of Z-transformed average pooled heterozygosity in ZBZ population (*Z*(*H*_P_)_ZBZ_) and wild boar (*Z*(*H*_P_)_WB_) respectively, as well as average fixation index (*Z*(*F*_ST_)), for autosomal 100 kb windows. b, The positive end of the *Z*(*F*_ST_) and the negative end of the *Z*(*H*_P_) distribution plotted along pig autosomes 1-18. A dashed horizontal line indicates the cut-off (|*Z*| > 2) used for extracting outliers.

**
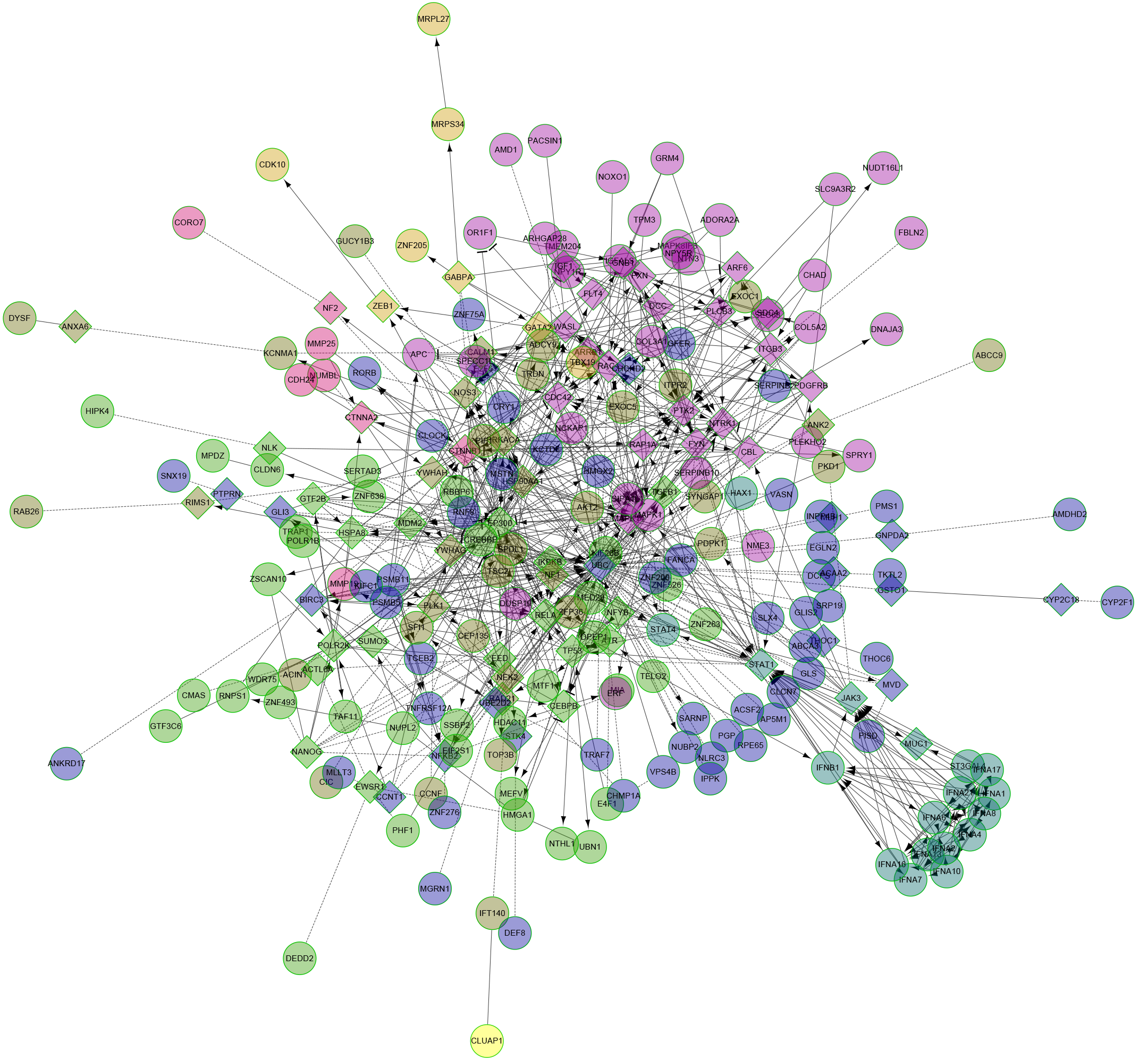
**

Supplementary Figure S9. Concise functional interaction network of CSGs. The effect of the interaction is represented by arrows, bar-headed lines, straight line and imaginary line.  "->" for activating/catalyzing, "-|" for inhibition, "-" for FIs extracted from complexes or inputs, and "---" for predicted FIs.

## Supplementary Table S1. The information of the downloaded genome data.

| **Breed** | **Accession No.** | **High-quality data (Gb)** | **References** |
| --- | --- | --- | --- |
| Chinese wild boar | SRS387324 | 16.28 | Li *et al*. (2013) |
|  | SRS387323 | 16.30 | Li *et al*. (2013) |
|  | SRS387320 | 12.00 | Li *et al*. (2013) |
|  | SRS465721 | 56.67 | Ai *et al*. (2015) |
|  | SRS465720 | 54.76 | Ai *et al*. (2015) |
|  | SRS465719 | 55.92 | Ai *et al*. (2015) |
|  | SRS465718 | 71.18 | Ai *et al*. (2015) |
|  | SRS465717 | 72.68 | Ai *et al*. (2015) |
|  | SRS465716 | 66.13 | Ai *et al*. (2015) |
|  | ERR173222 | 19.29 | Groenen *et al*. (2012) |
|  | ERR173221 | 9.31 | Groenen *et al*. (2012) |
|  | ERR173220 | 19.83 | Groenen *et al*. (2012) |
|  | ERR173219 | 9.83 | Groenen *et al*. (2012) |
| Korean wild boar | SRS703307 | 44.80 | Moon *et al*. (2015) |
|  | SRS703308 | 42.70 | Moon *et al*. (2015) |
|  | SRS703309 | 42.30 | Moon *et al*. (2015) |
|  | SRS703310 | 42.10 | Moon *et al*. (2015) |
|  | SRS703311 | 42.40 | Moon *et al*. (2015) |
|  | SRS703312 | 40.80 | Moon *et al*. (2015) |
|  | SRS703313 | 44.00 | Moon *et al*. (2015) |
|  | SRS703314 | 43.80 | Moon *et al*. (2015) |
|  | SRS703315 | 43.80 | Moon *et al*. (2015) |
|  | SRS703316 | 43.40 | Moon *et al*. (2015) |
| Luchuan | SRS465722 | 58.22 | Ai *et al*. (2015) |
|  | SRS465723 | 66.57 | Ai *et al*. (2015) |
|  | SRS465724 | 66.62 | Ai *et al*. (2015) |
|  | SRS465725 | 45.05 | Ai *et al*. (2015) |
|  | SRS465726 | 69.37 | Ai *et al*. (2015) |
|  | SRS465727 | 61.17 | Ai *et al*. (2015) |
| Wuzhishan | SRS465708 | 43.95 | Ai *et al*. (2015) |
|  | SRS465709 | 64.97 | Ai *et al*. (2015) |
|  | SRS465710 | 63.37 | Ai *et al*. (2015) |
|  | SRS465711 | 63.21 | Ai *et al*. (2015) |
|  | SRS465712 | 64.63 | Ai *et al*. (2015) |
|  | SRS465713 | 66.40 | Ai *et al*. (2015) |
| Xiang | ERR173223 | 18.40 | Groenen *et al*. (2012) |
|  | ERR173224 | 18.10 | Groenen *et al*. (2012) |
| Bamaxiang | SRS465762 | 65.39 | Ai *et al*. (2015) |
|  | SRS465763 | 69.52 | Ai *et al*. (2015) |
|  | SRS465764 | 63.44 | Ai *et al*. (2015) |
|  | SRS465765 | 67.23 | Ai *et al*. (2015) |
|  | SRS465766 | 66.30 | Ai *et al*. (2015) |
|  | SRS465767 | 66.33 | Ai *et al*. (2015) |
| Min | SRS465757 | 62.69 | Ai *et al*. (2015) |
|  | SRS465758 | 64.75 | Ai *et al*. (2015) |
|  | SRS465759 | 57.53 | Ai *et al*. (2015) |
|  | SRS465760 | 66.17 | Ai *et al*. (2015) |
|  | SRS465761 | 65.09 | Ai *et al*. (2015) |
| Erhualian | SRS465714 | 67.64 | Ai *et al*. (2015) |
|  | SRS465715 | 65.58 | Ai *et al*. (2015) |
|  | SRS465774 | 104.30 | Ai *et al*. (2015) |
|  | SRS465775 | 100.10 | Ai *et al*. (2015) |
|  | SRS465776 | 100.50 | Ai *et al*. (2015) |
| Hetao | SRS465750 | 63.71 | Ai *et al*. (2015) |
|  | SRS465751 | 60.96 | Ai *et al*. (2015) |
|  | SRS465752 | 51.76 | Ai *et al*. (2015) |
|  | SRS465753 | 59.89 | Ai *et al*. (2015) |
|  | SRS465754 | 62.35 | Ai *et al*. (2015) |
|  | SRS465755 | 61.12 | Ai *et al*. (2015) |
| Jinhua | SRS387315 | 11.07 | Li *et al*. (2013) |
|  | SRS387317 | 12.40 | Li *et al*. (2013) |
|  | SRS387319 | 10.60 | Li *et al*. (2013) |
| Laiwu | SRS465768 | 62.54 | Ai *et al*. (2015) |
|  | SRS465769 | 58.77 | Ai *et al*. (2015) |
|  | SRS465770 | 67.70 | Ai *et al*. (2015) |
|  | SRS465771 | 65.52 | Ai *et al*. (2015) |
|  | SRS465772 | 66.61 | Ai *et al*. (2015) |
|  | SRS465773 | 66.12 | Ai *et al*. (2015) |
| Meishan | ERS804949 | 30.80 | Frantz *et al*. (2015) |
|  | ERS804950 | 32.10 | Frantz *et al*. (2015) |
|  | ERS804953 | 23.80 | Frantz *et al*. (2015) |
|  | ERS804954 | 26.90 | Frantz *et al*. (2015) |
|  | ERS804955 | 32.30 | Frantz *et al*. (2015) |
|  | ERS804957 | 27.60 | Frantz *et al*. (2015) |
|  | ERS804951 | 31.10 | Frantz *et al*. (2015) |
|  | ERS804952 | 30.60 | Frantz *et al*. (2015) |
|  | ERS804956 | 16.30 | Frantz *et al*. (2015) |
|  | ERS804958 | 45.30 | Frantz *et al*. (2015) |
| Neijiang | SRS387304 | 15.70 | Li *et al*. (2013) |
|  | SRS387306 | 17.30 | Li *et al*. (2013) |
|  | SRS387311 | 11.40 | Li *et al*. (2013) |
| Penzhou | SRR652348 | 11.80 | Li *et al*. (2013) |
|  | SRR652349 | 11.80 | Li *et al*. (2013) |
|  | SRR652350 | 14.10 | Li *et al*. (2013) |
| Rongchang | SRS518091 | 19.07 | / |
|  | SRS518092 | 21.33 | / |
|  | SRS518093 | 24.90 | / |
|  | SRS518094 | 25.23 | / |
|  | SRS518095 | 26.15 | / |
|  | SRS518096 | 27.22 | / |
| Wujin | SRR652351 | 15.90 | Li *et al*. (2013) |
|  | SRR652352 | 14.20 | Li *et al*. (2013) |
|  | SRR652353 | 12.00 | Li *et al*. (2013) |
|  | SRS387298 | 12.10 | Li *et al*. (2013) |
|  | SRS387299 | 11.10 | Li *et al*. (2013) |
|  | SRS387302 | 13.10 | Li *et al*. (2013) |

## Supplementary Table S2. Summary of sequencing data.

| **population** | **Raw data (Gb)** | **High-quality base (Gb)** | **High-quality base** **ratio (%)** | **Mapping ratio (%)** | **Unique Mapping ratio (%)** | **Coverage at least 1 × (%)** | **Coverage at least 4 × (%)** |
| --- | --- | --- | --- | --- | --- | --- | --- |
| LCP | 70.64 | 64.85 | 91.81 | 93.32 | 82.17 | 81.30 | 74.85 |
| YTL | 67.86 | 61.99 | 91.35 | 93.37 | 82.45 | 81.47 | 75.09 |
| ZBZ | 63.71 | 60.25 | 94.56 | 93.80 | 82.97 | 81.95 | 77.08 |
| Wild boar | / | 61.82 | / | 96.65 | 87.44 | 82.54 | 78.87 |

LCP, Lvcongpo population (female, *n* = 31); YTL, Yetinglu population (female, *n* = 32); ZBZ, Zhongbaozhen population (female, *n* = 12). “Mapping ratio (%)” is percent of high-quality reads aligned on the pig reference genome (version 10.2). “Unique mapping ratio (%)” is percent of reads uniquely aligned over all the aligned reads.

## Supplementary Table S3. The number of SNPs identified in four different populations.

|  | **LCP** | **YTL** | **ZBZ** | **Wild boar** |
| --- | --- | --- | --- | --- |
| SNPs identified by SAMtools | 15,680,614 | 15,767,759 | 15,881,111 | 16,611,188 |
| SNPs identified by GATK | 16,061,496 | 16,050,452 | 16,593,507 | 16,234,465 |
| SNPs concurrently identified | 13,878,496 | 13,886,995 | 14,308,488 | 14,268,327 |
| *Known SNPs* | 13,595,465 | 13,608,456 | 14,013,250 | 13,977,569 |
| *Novel SNPs* | 283,031 | 278,539 | 295,238 | 290,758 |
| *Novel ratio (%)* | 2.04 | 2.02 | 2.06 | 2.04 |

LCP, Lvcongpo population; YTL, Yetinglu population; ZBZ, Zhongbaozhen population.

## Supplementary Table S4. Functional gene categories enriched for genes affected by nonsynonymous SNPs.

| **Term ID** | **Term description** | **Gene count** | ***P* value** |
| --- | --- | --- | --- |
| GO-BP:0007608 | Sensory perception of smell | 12 | 1.51×^-09^ |
| GO-BP:0007606 | Sensory perception of chemical stimulus | 37 | 4.70×^-26^ |
| GO-BP:0007600 | Sensory perception | 50 | 7.08×^-18^ |
| GO-BP:0050877 | Neurological system process | 63 | 3.57×^-10^ |
| GO-BP:0003008 | System process | 66 | 5.94×^-08^ |
| GO-BP:0044707 | Single-multicellular organism process | 70 | 1.84×^-05^ |
| GO-BP:0032501 | Multicellular organismal process | 70 | 2.02×^-05^ |
| GO-BP:0050896 | Response to stimulus | 83 | 1.10×^-04^ |
| GO-BP:0044238 | Primary metabolic process | 111 | 8.81×^-03^ |
| GO-BP:0008152 | Metabolic process | 129 | 4.37×^-05^ |

The *P* value was corrected by Benjamini method.

## Supplementary Table S5. Sequences of primers used for complete mtDNA amplification.

| **Primer ID** | **Forward primer sequence (5' to 3')** | **Reverse primer sequence (5' to 3')** |
| --- | --- | --- |
| **1** | GAGAATGCCCTCCAGATC | GGTTCAAAGTACCCATAT |
| **2** | AATACCCACCATACGAAA | CCTTTGCACGGTCAGAAT |
| **3** | GAGTAACAAGAAGCCTTT | GGGTATTGGTAGTGGAAC |
| **4** | CTCACCCTAGTAGAACGA | GGTAGTGTAGATAATGGGATTT |
| **5** | AGGTTCAAACCCTCTTAT | AGGAGTAGGCTAGTCGTA |
| **6** | TATCCCATACATGAAACAAG | CAAGGTGTAGGGAGAAAA |
| **7** | GACTCGTACCGCTAATA | GACATCCGTGTAGTCATT |
| **8** | TAGGCTCATTCATCTCAC | TCTGGGCTTGCTGGGTAT |
| **9** | AACCAAGCATGAGCAAAA | GCATACCATTGAGGGGAG |
| **10** | ATCCCAGGACGACTAAAC | CTGATAGGGCTCCGGTAA |
| **11** | ACAGCCAACATTACAGCA | CGATTAGTACGAGTAGGGA |
| **12** | TAATCATCGGATCTACTTTC | TCGGGTTGTGGTTTCTTT |
| **13** | TCTCCGACTCACTATCAG | TAATGTGGTGGGTGTATT |
| **14** | CCCCATCCATCAATCTAA | GTAATGCTGATACGGGAG |
| **15** | ATCGGATGATGACACGG | ATGTTGGTAGGCGGTGT |
| **16** | AACAGCCCTAATCGTAAC | CCTAAGAGGGAACCGAAG |
| **17** | TCCACCACTTACAATCAA | ATGGGTGTTCTACGGGTT |
| **18** | TATTCGCCTACGCTATCC | GCTGAGTCCAAGCATCCC |
| **19** | CGCGTGAAACCAGCAACC | TGGCACGAGATTTACCAACT |

## Supplementary Table S6. Information on the mitochondrial genomes used in this study.

| **Breed** | **­­­­Abbreviation** | **Accession number** | **References** |
| --- | --- | --- | --- |
| Enshi black pig | Enshi (ZBZ) | KX620009 | This study |
|  | Enshi (ZBZ) | KX620011 | This study |
|  | Enshi (LCP) | KX620010 | This study |
|  | Enshi (YTL) | KX620008 | This study |
|  | Enshi (YTL) | KX620012 | This study |
| Chinese Domestic | CD (Neijiang) | KC505406 | Li *et al*. (2014) |
|  | CD (Penzhou) | KC505407 | Li *et al*. (2014) |
|  | CD (Wujin) | KC505408 | Li *et al*. (2014) |
|  | CD (Yanan) | KC505409 | Li *et al*. (2014) |
|  | CD (Hainan) | AF486867 | Yang *et al*. (2003) |
|  | CD (Jinhua) | AF486863 | Yang *et al*. (2003) |
|  | CD (Yunan) | EF545574 | Wu *et al*. (2007) |
|  | CD (Shandong) | EF545589 | Wu *et al*. (2007) |
| Chinese Wild boar | CWB (Chongqing) | KC505411 | Li *et al*. (2014) |
|  | CWB (Fujian) | EF545569 | Wu *et al*. (2007) |
|  | CWB (Yunnan) | EF545585 | Wu *et al*. (2007) |
|  | CWB (Jilin) | EF545580 | Wu *et al*. (2007) |
|  | CWB (Hainan) | EF545572 | Wu *et al*. (2007) |
| European Domestic | ED (Duroc)* | / | Groenen *et al*. (2012) |
|  | ED (Landrace) | AF034253 | Lin *et al*. (1999) |
| European Wild boar | EWB (Italian) | AF304201 | Kijas & Andersson (2001) |
|  | EWB (Swedish) | AF304203 | Groenen *et al*. (2012) |
| Outgroup | Outgroup (Warthog) | NC_008830 | Wu *et al*. (2007) |

*, this mitochondrial genomes was extracted from Sscrofa10.2 reference sequence.
